# PDGFRα up-regulation mediated by sonic hedgehog pathway activation leads to BRAF inhibitor resistance in melanoma cells with BRAF mutation

**DOI:** 10.18632/oncotarget.1878

**Published:** 2014-03-31

**Authors:** Francesco Sabbatino, Yangyang Wang, Xinhui Wang, Keith T. Flaherty, Ling Yu, David Pepin, Giosue' Scognamiglio, Stefano Pepe, John M. Kirkwood, Zachary A. Cooper, Dennie T. Frederick, Jennifer A. Wargo, Soldano Ferrone, Cristina R. Ferrone

**Affiliations:** ^1^ Department of Surgery, Massachusetts General Hospital, Boston, MA; ^2^ Department of Medical Oncology, Massachusetts General Hospital, Boston, MA; ^3^ Harvard Medical School, Boston, MA; ^4^ Department of Clinical and Molecular Oncology and Endocrinology, University of Naples “Federico II”, Naples, Italy; ^5^ Department of Surgery, University of Pittsburgh Cancer Institute, University of Pittsburgh, Pittsburgh, Pittsburgh, PA; ^6^ Department of Medicine, University of Pittsburgh Cancer Institute, University of Pittsburgh, Pittsburgh, Pittsburgh, PA; ^7^ Institute for Clean Energy & Advanced Materials, Southwest University, Chongqing, P.R. China; ^8^ Pathology Unit, Istituto Nazionale Tumori Fondazione G. Pascale, Naples, Italy; ^9^ Department of Surgical Oncology, University of Texas MD Anderson Cancer Center, Houston, TX

**Keywords:** BRAF inhibitor resistance, PDGFRα up-regulation, PDGFRα inhibitors, melanoma, sonic hedgehog pathway, LDE225

## Abstract

Control of BRAF(V600E) metastatic melanoma by BRAF inhibitor (BRAF-I) is limited by intrinsic and acquired resistance. Growth factor receptor up-regulation is among the mechanisms underlying BRAF-I resistance of melanoma cells. Here we demonstrate for the first time that PDGFRα up-regulation causes BRAF-I resistance. PDGFRα inhibition by PDGFRα-specific short hairpin (sh)RNA and by PDGFRα inhibitors restores and increases melanoma cells' sensitivity to BRAF-I *in vitro* and *in vivo*. This effect reflects the inhibition of ERK and AKT activation which is associated with BRAF-I resistance of melanoma cells. PDGFRα up-regulation is mediated by Sonic Hedgehog Homolog (Shh) pathway activation which is induced by BRAF-I treatment. Similarly to PDGFRα inhibition, Shh inhibition by LDE225 restores and increases melanoma cells' sensitivity to BRAF-I. These effects are mediated by PDGFRα down-regulation and by ERK and AKT inhibition. The clinical relevance of these data is indicated by the association of PDGFRα up-regulation in melanoma matched biopsies of BRAF-I +/- MEK inhibitor treated patients with shorter time to disease progression and less tumor regression. These findings suggest that monitoring patients for early PDGFRα up-regulation will facilitate the identification of those who may benefit from the treatment with BRAF-I in combination with clinically approved PDGFRα or Shh inhibitors.

## Introduction

Over 50% of metastatic melanomas harbor the BRAF(V600E) point mutation (T1799A)[1, 2]. Mutant BRAF(V600E) represents a constitutively active protein serine kinase that leads to the sustained activation of the BRAF→MEK1/2→ERK1/2 MAP kinase pathway[3, 4]. This pathway plays a critical role in the regulation of gene expression as well as cell proliferation and survival, which are all involved in the initiation and progression of melanoma[5, 6]. Clinical trials have demonstrated that the BRAF inhibitor (BRAF-I), PLX4032 (vemurafenib), and other inhibitors in its class (GSK2118436 or dabrafenib) can induce tumor regression in more than 50% of the patients with metastatic melanoma harboring the BRAF(V600E) mutation and improve both progression-free and overall survival [7, 8]. Although the clinical activity of BRAF-I therapy is a major breakthrough in the treatment of metastatic melanoma, the median time to disease progression is less than 7 months due to acquired resistance[8]. Furthermore complete responses to vemurafenib are only observed in 5% of patients, as a consequence of intrinsic BRAF-I resistance[7, 9].

The multiple mechanisms underlying melanoma BRAF-I resistance, most of which have been validated by clinical evidence[10-14], can be classified into two groups. One includes ERK signaling reactivation, caused by point mutations in MEK1[10, 15], amplification of mutant BRAF(V600E)[16], elevated CRAF activity[17], activating NRAS mutations [11], increased levels of COT/Tpl2[12] and/or aberrantly spliced BRAF(V600E)[13]. The other one includes activation of alternative pro-tumorigenic pathways such as the PI3K/AKT pathway that can be caused by phosphatase and tensin homolog (PTEN) loss [18] or by an increase in signaling driven by receptor tyrosine kinases (RTK). The latter include the platelet-derived growth factor (PDGFR)β [11, 19] and the insulin-like growth factor receptor (IGF1R) [14]. To the best of our knowledge, PDGFRα, a RTK which markedly differs in its functional properties from its family member PDGFRβ, has not been implicated in BRAF-I resistance of BRAF(V600E) melanoma.

In this study we provide for the first time both *in vitro* and *in vivo* evidence that PDGFRα up-regulation causes BRAF-I resistance of BRAF(V600E) melanoma cells. Furthermore, we show that PDGFRα up-regulation is mediated by activation of the Sonic Hedgehog Homolog (Shh) pathway which is induced by BRAF-I treatment. Lastly, we describe combinatorial strategies which can be easily translated to a clinical setting to counteract the Shh/PDGFRα mediated BRAF-I resistance of BRAF(V600E) melanoma cells.

## Results

### ERK reactivation, AKT activation and PDGFRα up-regulation in melanoma cell lines with acquired BRAF-I resistance

**Figure 1:**
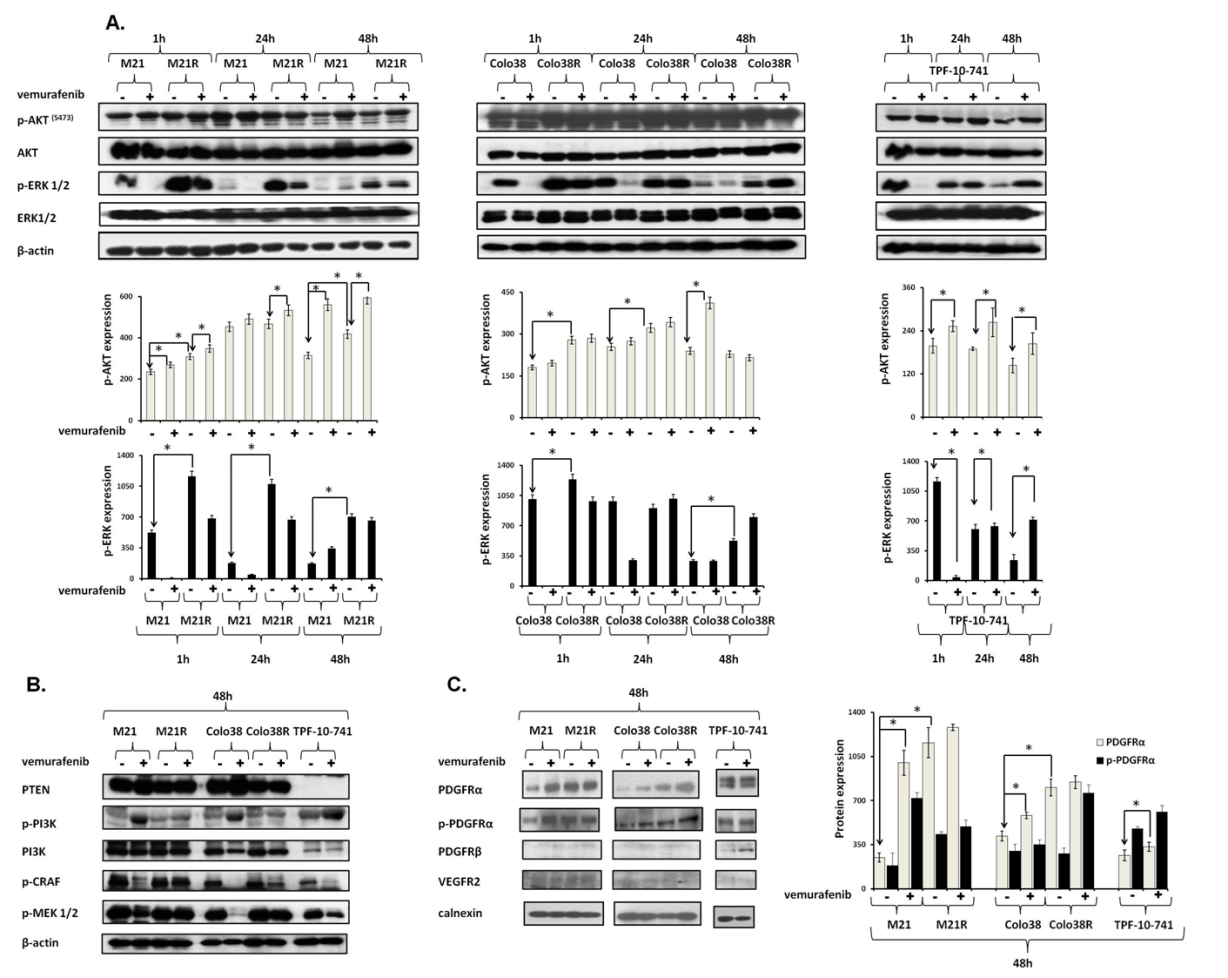
Association of BRAF-I resistance with MAPK reactivation, PI3K/AKT activation and PDGFRα up-regulation in BRAF(V600E) melanoma cell lines.Cells were treated with the BRAF-I vemurafenib (1 μM) A. Following an up to 48 h incubation at 37°C cells were harvested and lysed. Cell lysates were analyzed by western blot with the indicated mAbs. β-actin was used as a loading control. A representative result is shown (upper panel). The levels of p-ERK and p-AKT normalized to β-actin are plotted and expressed as mean ± SD of the results obtained in three independent experiments (lower panel). The asterisk (*) indicates *P*<0.05. B. Following a 48 h incubation at 37°C cells were harvested and lysed. Cell lysates were analyzed by western blot with the indicated mAbs. β-actin was used as a loading control. C. Following a 48 h incubation at 37°C cells were harvested and lysed. Cell lysates were analyzed by western blot with the indicated mAbs. Calnexin was used as a loading control. A representative result is shown (left panel). The levels of PDGFRα and p-PDGFRα normalized to calnexin are plotted and expressed as mean ± SD of the results obtained in three independent experiments (right panel). The asterisk (*) indicates *P*<0.05.

The parental Colo38 and M21 cell lines were compared in their sensitivity to the anti-proliferative activity of the BRAF-I vemurafenib to the autologous cell lines Colo38R, and M21R and the allogeneic cell line TPF-10-741. Parental Colo38 and M21 cells were highly sensitive to the anti-proliferative activity of vemurafenib at the concentrations ranging between 250 nM and 2000 nM. In contrast, Colo38R and M21R cells showed a markedly lower sensitivity to the growth inhibitory effects of vemurafenib (Supplementary Figure 1). TPF-10-741 cells displayed an intermediate sensitivity to vemurafenib. This acquired resistance model was used to investigate the molecular mechanisms underlying disease progression after an initial response to vemurafenib. Since acquired BRAF-I resistance can be mediated by reactivation of the MAPK pathway or by activation of alternative pathways like PI3K/AKT, we evaluated signaling through these pathways in both parental and resistant cell lines (Figure 1A). Following a 1 and a 24 hour (h) incubation at 37°C with vemurafenib, phospho- (p)-ERK levels were markedly reduced in both Colo38 and M21 cells, but were changed to a limited extent or not at all in Colo38R and M21R cells. The latter cells also displayed much higher levels of p-ERK as compared to the parental cells under basal conditions (*P*<0.05). As described by Lito *et al*. in other BRAF(V600E) melanoma cell lines [20], p-ERK levels rebounded after a 24 h incubation at 37°C with vemurafenib in both Colo38 and M21 cells. However no changes were detected in Colo38R and M21R cells. Similarly to the resistant cells, in partially resistant TPF-10-741 cells p-ERK levels were changed to a limited extent rebounding just at 24 h incubation with vemurafenib. p-AKT levels were increased in Colo38R and M21R cells compared to Colo38 and M21 cells (*P*<0.05). p-AKT levels were also increased in Colo38, M21 and TPF-10-741 cells after treatment with vemurafenib (*P*<0.05).

To investigate the mechanisms underlying the melanoma cell resistance to BRAF-I, the expression and activation of the RAF/MEK/ERK and PI3K/AKT pathway components were analyzed in the cell lines both under basal conditions and after treatment with vemurafenib. CRAF and MEK were reactivated in Colo38R, M21R, and TPF-10-741 cells. PI3K was activated in Colo38, M21 and TPF-10-741 cells after treatment with vemurafenib (Figure 1B), but its levels were not affected in Colo38R and M21R cells. PTEN was not detected in TPF-10-741 cells, but was expressed in the other cell lines (Figure 1B). Therefore the resistance of melanoma cell lines to BRAF-I was associated with the simultaneous reactivation of the MAPK pathway and with the activation of the PI3K/AKT pathway. The latter might be caused by an upstream activator of MAPK and PI3K/AKT pathways. To exclude the presence of NRAS mutations as well as the presence of additional alterations in BRAF gene, a RT-PCR was performed in parental and resistant cell lines. No changes were detected in NRAS sequence and the BRAF(V600E) mutation was present in both parental and resistant cell lines (Supplementary Figure S2). Since BRAF-I resistance through reactivation of MAPK pathway and activation of PI3K/AKT pathway can be mediated by RTK up-regulation [11], we investigated the potential role of RTK PDGFRα, PDGFRβ and VEGFR2 in BRAF-I resistance. As shown in Figure 1C, vemurafenib enhanced PDGFRα expression and activation in Colo38 and M21 cells (*P*<0.05). Furthermore PDGFRα levels were higher in Colo38R and M21R cells as compared to the parental cells (*P*<0.05). PDGFRα was expressed and activated in TPF-10-741 cells both under basal conditions and following treatment with vemurafenib. PDGFRβ was up-regulated on TPF-10-741 cells after treatment with vemurafenib, but was not detectable in the other cell lines both under basal conditions and following treatment with vemurafenib. Lastly, VEGFR2 expression was not detected in any cell lines before or after treatment with vemurafenib.

### Induction by PDGFRα up-regulation of melanoma cell line resistance to BRAF-I

**Figure 2:**
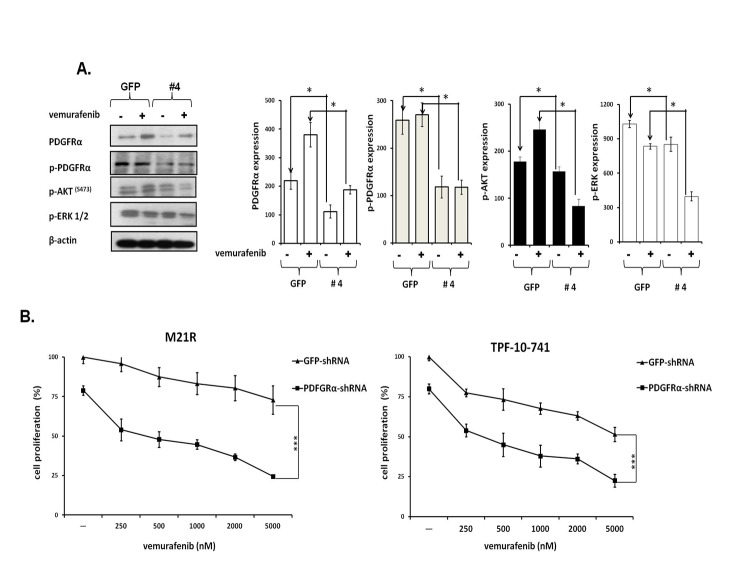
Restoration by PDGFRα down-regulation of BRAF-I sensitivity of BRAF(V600E) melanoma cell lines with acquired BRAF-I resistance A. M21R transduced with PDGFRα-specific shRNA (#4) or GFP-shRNA lentiviral particles were treated with the BRAF-I vemurafenib (1μM). Following a 3 day incubation at 37°C cells were harvested and lysed. Cell lysates were analyzed by western blot with the indicated mAbs. β-actin was used as a loading control. A representative result is shown (left panel). The levels of PDGFRα, p- PDGFRα, p-ERK and p-AKT normalized to β-actin are plotted and expressed as mean ± SD of the results obtained in three independent experiments (right panel). The asterisk (*) indicates *P*<0.05. B. PDGFRα-specific shRNA (#4) transduced M21R and TPF-10-741 cells were treated with the indicated vemurafenib concentrations. GFP-shRNA transduced M21R and TPF-10-741 cells were used as controls. Cell proliferation was determined by MTT assay following a 3 day incubation at 37°C. Percentage of cell proliferation was calculated as the ratio of treated cells to untreated GFP-shRNA transduced cells. Data are expressed as mean ± SD of the results obtained in three independent experiments. The asterisks (***) indicate *P*<0.01.

To test whether PDGFRα up-regulation caused BRAF-I resistance in Colo38R, M21R and TPF-10-741 cells, PDGFRα was knocked down in the three cell lines using 5 PDGFRα-specific short hairpin RNA (shRNAs). As shown in Figure 2A, lentiviral transduction of M21R cells with a PDGFRα-specific shRNA(#4) construct knocked down p-PDGFRα and PDGFRα expression (p<0.05). PDGFRα down-regulation was associated with a minimal decrease in p-ERK and p-AKT levels (*P*<0.05) as compared to untreated cells. However, this effect was markedly enhanced when the cells transduced with PDGFRα-specific shRNA(#4) were treated with vemurafenib (*P*<0.05) which slightly decreased p-ERK levels and increased p-AKT expression. Additionally, as shown in Figure 2B, M21R and TPF-10-741 cells transduced with the PDGFRα-specific shRNA(#4) displayed a significantly increased sensitivity to the anti-proliferative effect of vemurafenib when compared to the autologous cells transduced with a GFP-shRNA (*P*<0.01) (IC50 ≤ 20 times).

### Association of PDGFRα up-regulation in melanoma patient derived biopsies with BRAF-I resistance

**Figure 3:**
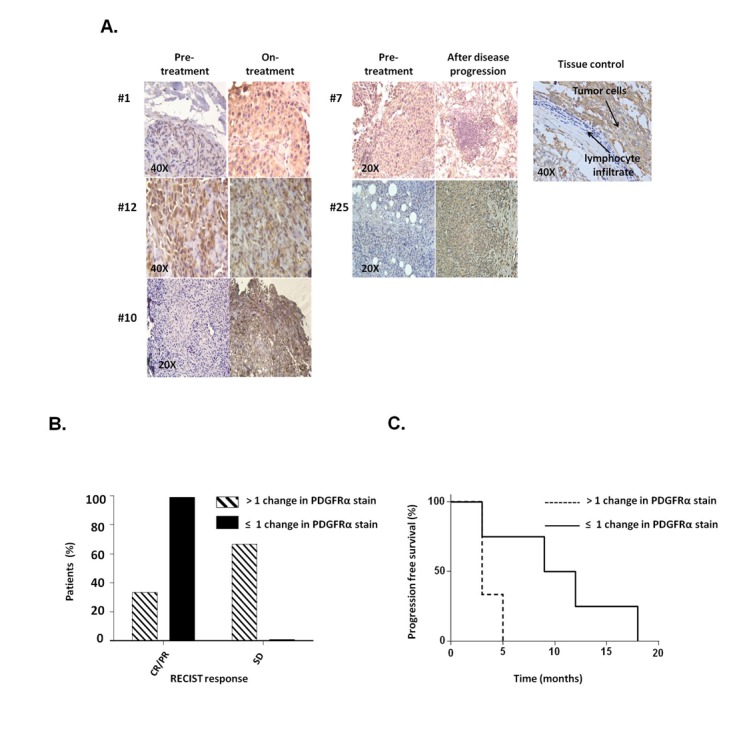
PDGFRα expression in melanoma metastases obtained from patients who acquired BRAF-I resistance Melanoma tumors were biopsied before treatment (day 0), at 10-14 days on treatment, and/or at the time of disease progression following treatment with BRAF-I or with BRAF-I and MEK-I. Tumor sections were stained with H&E and PDGFRα-specific rabbit antibody. Scores were recorded semiquantitatively as 1+, 2+, 3+ and 4+, when 1–25%, 26–50%, 51-75% and >75% of melanoma cells were stained, respectively. Patients were divided in two groups based on change of PDGFRα expression, as measured by IHC staining of melanoma biopsies: those whose PDGFRα staining score had no or 1 point increase after treatment (≤1+) and those whose PDGFRα staining score increased 2 or more points after treatment (>1+). A. Representative IHC staining of PDGFRα expression in melanoma patients before treatment, at 10-14 days on treatment and at the time of disease progression in 5 out of 9 tumor biopsies. Tissue from a human GIST and its lymphocyte infiltrate were used as a positive and a negative control, respectively, for PDGFRα expression. The magnification used is indicated in the panels of the figure. B. Two groups of patients were graphed based upon RECIST (complete response (CR), partial response (PR) and stable disease (SD)) and compared as a percent of the total population of the PDGFRα stain score group. C. Two groups of patients were graphed based upon the time to disease progression utilizing Kaplan–Meier method.

To assess the potential clinical significance of our *in vitro* results, we tested PDGFRα expression in biopsies obtained from 9 melanoma patients treated with BRAF-I or with the novel combination of BRAF-I and MEK inhibitor (MEK-I) [21]. Tumor biopsies were performed pre-treatment (day 0), at 10-14 days on treatment, and/or at the time of disease progression. Immunohistochemical (IHC) staining demonstrated PDGFRα up-regulation in 5 out of 9 patients following treatment with BRAF-I +/- MEK-I (Figure 3A). In 3 of the 5 patients a significant increase in PDGFRα expression (>1+) was observed after treatment. Patients with a significant (>1+) increase in PDGFRα expression after treatment with BRAF-I +/- MEK-I had less tumor regression (Figure 3B) and shorter time to disease progression (Figure 3C) (*P*=0.07) when compared to patients who had no change or a small change in PDGFRα expression (≤1+).

### Increase by PDGFRα-I of the anti-tumor activity of BRAF-I in BRAF-I sensitive and resistant melanoma cell lines

**Figure 4:**
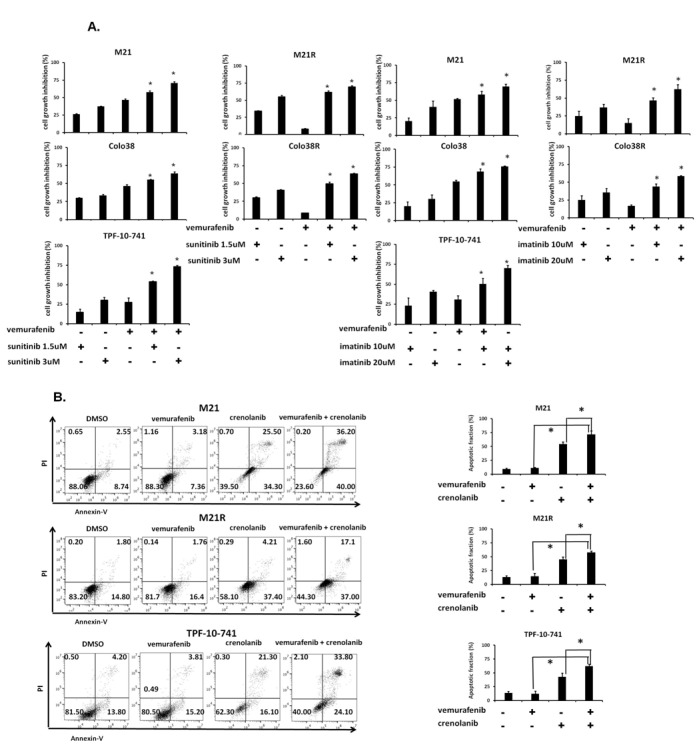
Enhancement by PDGFRα-I of the *in vitro* anti-proliferative and pro-apoptotic activity of BRAF-I in BRAF-I sensitive and resistant melanoma cell lines harboring BRAF(V600E) A. Cells were treated with the BRAF-I vemurafenib (500 nM) and/or the indicated concentration of PDGFRα-I sunitinib (left panel) or imatinib (right panel). Cell growth inhibition was determined by MTT assay following a 3 day incubation at 37°C. Percentage of cell growth inhibition was calculated as ratio of treated to untreated cells for each treatment. Data are expressed as mean ± SD of the results obtained in three independent experiments. The asterisk (*) indicates *P*<0.05. B. Cells were treated with the BRAF-I vemurafenib (500 nM) and/or the PDGFRα-I crenolanib (1 μM). Following a 24 h incubation at 37°C cells were harvested and stained with Annexin V and PI. A representative result is shown (left panel). The levels of apoptosis are plotted and expressed as mean fraction of apoptotic cells ± SD of the results obtained in three independent experiments (right panel). The asterisk (*) indicates *P*<0.05.

To investigate whether the anti-tumor activity of BRAF-I could be enhanced by PDGFRα inhibition, Colo38, Colo38R, M21, M21R and TPF-10-741 cells were treated with vemurafenib in combination with the PDGFRα inhibitor (PDGFRα-I) sunitinib [22], imatinib [23] or crenolanib [24]. A titration experiment established the dose of the PDGFRα-I to be combined with vemurafenib in the 5 cell lines. The IC50 doses of sunitinib, imatinib and crenolanib were found to be 2, 15 and 1.5 μM, respectively (Supplementary Figure 3). In line with the data in the literature [25-35], the doses of 1.5 and 3 μM for sunitinib, 10 and 20 μM for imatinib and 1 and 2 μM for crenolanib, were tested in combination with vemurafenib for their anti-proliferative effect and induction of apoptosis. As shown in Figure 4A and Supplementary Figure 4, vemurafenib and PDGFRα-I combination inhibited the proliferation of Colo38 and M21 cells to a significantly greater extent (*P*<0.05) than each agent alone. Furthermore, as observed with cells transduced with the PDGFRα-specific shRNA, PDGFRα-I synergized (*P*<0.05) with vemurafenib in the inhibition of Colo38R, M21R and TPF-10-741 cell growth. Lastly, vemurafenib and PDGFRα-I (crenolanib or sunitinib) combination (Figure 4B and Supplementary Figure 5) induced apoptosis in a significantly (*P*<0.05) higher percentage of cells than each agent alone in both BRAF-I sensitive and resistant cell lines. It is worth noting that crenolanib and sunitinib induced apoptosis in both BRAF-I sensitive and resistant cell lines (*P*<0.05), while vemurafenib had no detectable effect.

### Inhibition by BRAF-I and PDGFRα-I of ERK and AKT activation in BRAF-I sensitive and resistant melanoma cell lines

**Figure 5:**
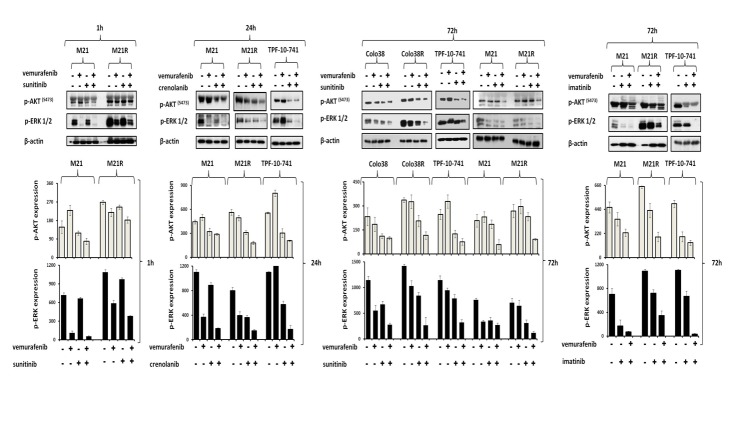
Enhancement by PDGFRα-I of the signaling pathway inhibition by BRAF-I in BRAF-I sensitive and resistant melanoma cell lines harboring BRAF(V600E) Cells were treated with the BRAF-I vemurafenib (1 μM) and/or the PDGFRα-I sunitinib (1.5 μM) and/or imatinib (10 μM) and/or crenolanib (1 uM). Following an up to 72 h incubation at 37°C, cells were harvested and lysed. Cell lysates were analyzed by western blot with the indicated mAbs. β-actin was used as a loading control. Representative results are shown (upper panel). The levels of p-ERK and p-AKT normalized to β-actin are plotted and expressed as mean ± SD of the results obtained in two independent experiments (lower panel).

We next investigated whether the enhanced anti-proliferative and pro-apoptotic activity of BRAF-I and PDGFRα-I combination was mediated by an increased inhibition of ERK and AKT activation in BRAF-I sensitive and resistant cells. As shown in Figure 5, p-ERK and p-AKT levels were markedly decreased in both BRAF-I sensitive and resistant melanoma cells after treatment with vemurafenib and PDGFRα-I combination. Specifically, p-ERK levels were dramatically decreased in Colo38 and M21 cells treated with vemurafenib. In contrast p-ERK levels were minimally decreased in Colo38 and M21 cells treated with PDGFRα-I. In addition, p-AKT levels were increased in M21 cells treated with vemurafenib, but were reduced in Colo38 and M21 cells treated with PDGFRα-I. However both p-ERK and p-AKT levels were markedly inhibited in Colo38 and M21 cells treated with vemurafenib and PDGFRα-I combination. On the other hand, p-ERK levels were minimally inhibited by vemurafenib in TPF-10-741 cells as well as in Colo38R and M21R cells when compared with parental cell lines. As observed with cells transduced with the PDGFRα-specific shRNA, PDGFRα-I decreased p-ERK and p-AKT levels in Colo38R, M21R and TPF-10-741 cells. However, vemurafenib and PDGFRα-I combination markedly decreased both p-ERK and p-AKT levels to a greater extent than each agent alone in all of the BRAF-I resistant cell lines (Figure 5).

### Enhancement by PDGFRα-I of the *in vivo* anti-tumor activity of BRAF-I in BRAF-I sensitive and resistant BRAF(V600E) melanoma cell lines

**Figure 6:**
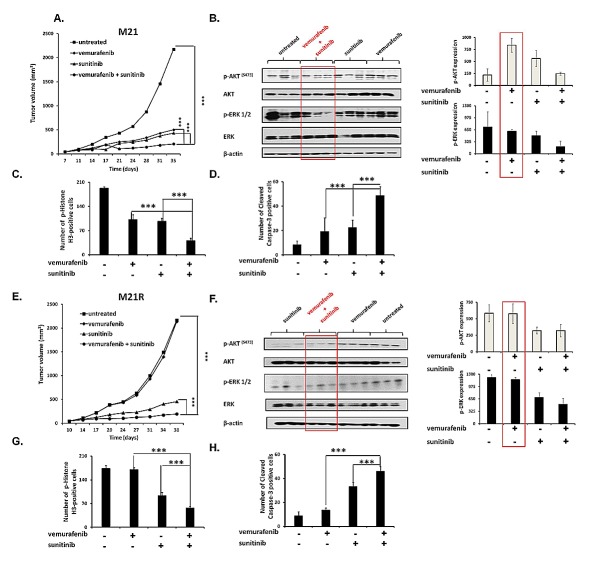
Enhancement by PDGFRα-I of the anti-tumor activity of BRAF-I in human BRAF(V600E) melanoma cells grafted in immunodeficient mice A, E. M21 and M21R cells were each implanted subcutaneously in 20 SCID mice. When tumors became palpable, mice were randomly divided into 4 groups (5 mice/group). One group was treated with the BRAF-I vemurafenib (12.5 mg/kg/twice per day), one with the PDGFRα-I sunitinib (20 mg/kg/day) and one with vemurafenib (12.5 mg/kg/twice per day) in combination with sunitinib (20 mg/kg/day). One group of mice was left untreated as a reference for the natural course of the disease. Efficacy data are plotted as mean tumor volume (in mm^3^) ± SD. The asterisks (***) indicate *P*<0.001. B, F. Tumors harvested from untreated and treated mice were lysed and analyzed for expression and activation of the indicated signaling pathway components. β-actin was used as a loading control. Three representative tumor cell lysates for each group of mice are shown (left panel). The levels of p-ERK and p-AKT normalized to β-actin are plotted and expressed as mean ± SD of the results obtained with the five tumor cell lysates in each group (right panel). C, G. Tissue sections obtained from the harvested tumors were analyzed for the content of mitotic cells by staining with p-Histone H3 (Ser10) protein-specific antibody. Mitotic tumor cells were quantified by counting 5 randomly selected high-power fields per section (magnification ×200). D, H. Tissue sections obtained from the harvested tumors were analyzed for the content of apoptotic cells by staining with Cleaved Caspase-3 (Asp175)-specific antibody. Apoptotic tumor cells were quantified by counting 5 randomly selected high-power fields per section (magnification ×200). Data are presented as means ± SD. The asterisks (***) indicate *P*<0.001.

To assess the *in vivo* relevance of our *in vitro* results, vemurafenib and sunitinib combination was tested for its ability to inhibit the growth of M21 and M21R cells in severe combined immunodeficiency (SCID) mice. The oral administration of the drugs, either in combination or as individual agents, caused no overt side effects (data not shown). In the mice grafted with M21 cells (Figure 6A) vemurafenib (12.5 mg/kg twice per day) and sunitinib (20 mg/Kg/day) combination inhibited tumor growth to a significantly (*P*<0.001) greater extent than each single agent. Both vemurafenib and sunitinib inhibited tumor growth as single agents to a similar extent (*P*<0.001). It is noteworthy that sunitinib was administered at a lower dose (20 mg/Kg/day) as compared to the dose used by other investigators (40 mg/Kg/day)[26, 27, 36]. Nevertheless, sunitinib was effective in enhancing the anti-tumor activity of vemurafenib. Analysis of the tumor cell lysates removed from treated and untreated mice (Figure 6B) demonstrated that vemurafenib and sunitinib combination markedly reduced both p-ERK and p-AKT levels. Sunitinib by itself slightly decreased p-ERK and p-AKT levels, while vemurafenib decreased p-ERK levels but increased p-AKT levels. IHC analysis of the primary tumors demonstrated a marked reduction in the number of mitoses in tumors from mice treated with vemurafenib and sunitinib combination (Figure 6C) when compared to tumors from mice treated with each single agent (*P*<0.001) or from untreated mice (*P*<0.001). Both vemurafenib and sunitinib reduced significantly the number of mitoses in tumors as compared to untreated mice (*P*<0.001). The number of apoptotic cells (Figure 6D) in tumors from mice treated with vemurafenib and sunitinib combination was significantly higher than in tumors from untreated mice or from mice treated with vemurafenib or sunitinib individually (*P*<0.001). Sunitinib, but not vemurafenib induced apoptosis in a significantly higher number of cells in tumors when compared to untreated mice (*P*<0.001).

In the mice grafted with M21R cells (Figure 6E), as expected, vemurafenib did not inhibit tumor growth as compared to untreated mice. In contrast, sunitinib significantly inhibited tumor growth as compared to untreated mice (*P*<0.001) or to mice treated with vemurafenib (*P*<0.001). This effect was significantly enhanced when sunitinib was combined with vemurafenib (*P*<0.001). Analysis of the tumor lysates (Figure 6F) demonstrated that, while vemurafenib had no detectable effect on p-ERK and p-AKT levels, sunitinib inhibited both of them. This effect was more marked in tumors from mice treated with vemurafenib and sunitinib combination. IHC analysis of the primary tumors showed a significantly lower number of mitoses in tumors from mice treated with sunitinib (Figure 6G) when compared to that in tumors from vemurafenib treated or untreated mice (*P*<0.001). In addition, sunitinib strongly increased the number of apoptotic cells in tumors as compared to vemurafenib or untreated mice (*P*<0.001) (Figure 6H). However vemurafenib and sunitinib combination decreased the number of mitotic cells and increased that of apoptotic cells to a significantly (*P*<0.001) greater extent than sunitinib alone.

To prove that the results obtained with sunitinib did not reflect potential off target effects of sunitinb we tested the therapeutic efficacy of the other PDGFRα-I imatinib in combination with a higher dose of vemurafenib (25 mg/kg twice per day). Imatinib was administered at a dose (100 mg/kg/day) which has been used by other investigators [31, 32]. As expected vemurafenib and imatinib combination inhibited tumor growth of M21 cells to a significantly (*P*<0.001) greater extent than each single agent, although imatinib displayed lower anti-tumor activity than sunitinib and vemurafenib had a higher anti-tumor effect. (Supplementary Figure 6).

### Role of Shh pathway activation in PDGFRα up-regulation mediated BRAF-I resistance of melanoma cells

**Figure 7:**
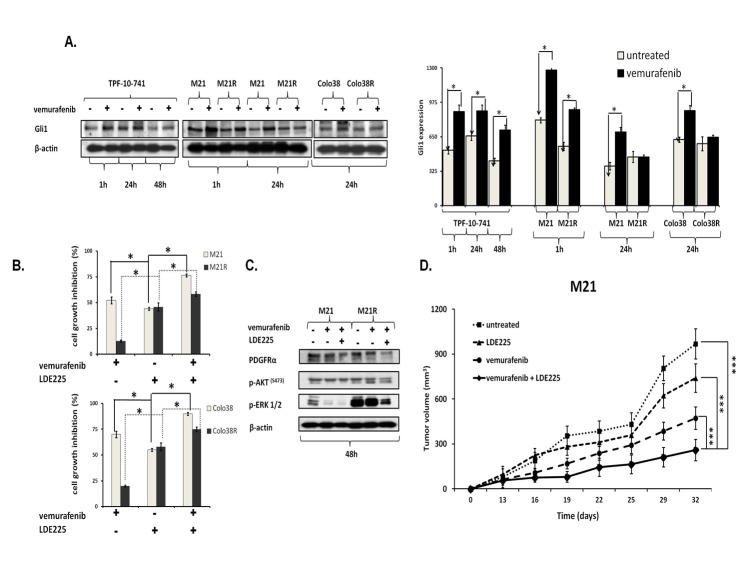
Association of Gli1 activation with PDGFRα up-regulation mediating BRAF-I resistance in melanoma cell lines harboring BRAF(V600E) A. Cells were treated with the BRAF-I vemurafenib (1 μM). Following an up to 72 h incubation at 37°C cells were harvested and lysed. Cell lysates were analyzed by western blot with the indicated mAbs. β-actin was used as a loading control. A representative result is shown (left panel). The level of Gli1 normalized to β-actin is plotted and expressed as mean ± SD of the results obtained in three independent experiments (right panel). The asterisk (*) indicates *P*<0.05. B. Cells were treated with vemurafenib (1 uM) and/or the Shh-I LDE225 (10 uM). Cell growth inhibition was determined by MTT assay following a 3 day incubation at 37°C. Percentage of cell growth inhibition was calculated as the ratio of treated to untreated cells. Data are expressed as the mean ± SD of the results obtained in three independent experiments. The asterisk (*) indicates *P*<0.05. C. M21 and M21R cells were treated with vemurafenib (1 μM) and/or LDE225 (10 uM). Following a 48 h incubation at 37°C cells were harvested and lysed. Cell lysates were analyzed by western blot with the indicated mAbs. β-actin was used as a loading control. The data shown are representative of the results obtained in two independent experiments. D. M21 cells were implanted subcutaneously in 20 SCID mice. When tumors became palpable, mice were randomly divided into 4 groups (5 mice/group). One group was treated with the BRAF-I vemurafenib (12.5 mg/kg/twice per day), one with the Shh-I LDE225 (40 mg/kg/day) and one with vemurafenib (12.5 mg/kg/twice per day) in combination with LDE225 (40 mg/kg/day). One group of mice was left untreated as a reference for the natural course of the disease. Efficacy data are plotted as mean tumor volume (in mm^3^) ± SD. The asterisks (***) indicate *P*<0.001.

The previously described role of the Shh pathway and Gli1 activation[37-40] in PDGFRα up-regulation associated with the MAPK and PI3K/AKT pathway activation prompted us to investigate whether Gli1 activation is involved in PDGFRα up-regulation in BRAF(V600E) melanoma cells. As shown in Figure 7A, vemurafenib enhanced Gli1 expression in Colo38, Colo38R, M21, M21R, and TPF-10-741 cells as compared to untreated cells (*P*<0.05). Similarly to PDGFRα inhibition, inhibition of Gli1 activation by the novel clinically available Shh inhibitor (Shh-I) LDE225[41] restored (*P*<0.05) and increased (*P*<0.05) melanoma cells' sensitivity to BRAF-I (Figure 7B). Furthermore, LDE225 in combination with vemurafenib down-regulated PDGFRα expression and inhibited ERK and AKT activation in the BRAF-I sensitive and resistant melanoma cells (Figure 7C). Lastly LDE225 (40 mg/Kg/day) enhanced (p<0.001) the ability of vemurafenib to inhibit the growth of M21 cells in SCID mice (Figure 7D). These results validated the association between Gli1 activation and PDGFRα up-regulation mediating BRAF-I resistance.

## Discussion

PDGFRα is overexpressed in sarcoma and glioma. It is involved in tumor growth, metastasis and neo angiogenesis, as well as in the development of resistance to cytotoxic therapy [42]. These functional properties of PDGFRα are likely to reflect its ability to engage signaling pathways, such as RAS/RAF/MEK/ERK and PI3K/AKT which play a role in tumor cell proliferation and aggressive phenotype. The present study demonstrates that human melanoma cells express PDGFRα both *in vitro* and *in vivo*. PDGFRα up-regulation in human melanoma cells harboring the BRAF(V600E) mutation is shown for the first time to be associated with the loss of their sensitivity to the anti-proliferative and pro-apoptotic activity of the BRAF-I vemurafenib both *in vitro* and *in vivo*. The association between PDGFRα up-regulation and vemurafenib resistance reflects a cause-effect relationship. Vemurafenib resistance is overcome in melanoma cells which down-regulate PDGFRα expression following transduction with a PDGFRα-specific shRNA. An association between the PDGFRα and BRAF(V600E) mutation is also observed in wild type PDGFRα gastrointestinal stromal tumors (GISTs) which acquire the BRAF(V600E) mutation when they develop resistance to PDGFRα-I imatinib [43-46].

Vemurafenib resistance of melanoma cells harboring a BRAF mutation reflects ERK and AKT activation induced by PDGFRα up-regulation, since inhibition of its synthesis by PDGFRα-specific shRNA causes a reduction of ERK and AKT activation and restores sensitivity to BRAF-I. A similar effect has been demonstrated for the HGF mediated resistance to BRAF-I [47]. This conclusion is corroborated by the *in vitro* and *in vivo* results obtained by inhibiting the function of PDGFRα with the clinically approved tyrosine kinase inhibitors sunitinib, imatinib and crenolanib. Sunitinib is an inhibitor of PDGFRα, PDGFRβ and VEGFR2. Imatinib is an inhibitor of PDGFRα, PDGFRβ. Crenolanib is a novel and potent inhibitor of PDGFRα and PDGFRβ. It is worth noting that the BRAF(V600E) melanoma cell lines with a PDGFRα up-regulation mediated BRAF-I resistance did not express PDGFRβ and VEGFR2. Vemurafenib and PDGFRα-I combination markedly inhibits *in vitro* proliferation and induces apoptosis of melanoma cells with a PDGFRα up-regulation mediated BRAF-I resistance. These results are paralleled by our *in vivo* findings. Vemurafenib and PDGFRα-I combination inhibited the growth and induced apoptosis in human melanoma cells with PDGFRα up-regulation mediated BRAF-I resistance engrafted in immunodeficient mice. These effects are mediated by the inhibition of the RAF/MEK/ERK and PI3K/AKT signaling pathways. The levels of p-ERK and p-AKT were markedly reduced in melanoma cells with PDGFRα up-regulation mediated BRAF-I resistance following *in vitro* or *in vivo* treatment with vemurafenib and PDGFRα-I combination. It is noteworthy that this combination has a significantly greater anti-proliferative and pro-apoptotic effect than either agent alone both *in vitro* and *in vivo* also with BRAF-I sensitive human melanoma cells which express PDGFRα. Therefore, our results suggest that the combinatorial strategy we have designed may overcome not only the acquired, but also the intrinsic BRAF-I resistance if PDGFRα is expressed. Furthermore they confirm that simultaneous inhibition of both the ERK and AKT pathways is more effective in suppressing tumor cell proliferation and in inducing apoptosis in both BRAF-I sensitive and resistant melanoma cells[14, 48-54].

In agreement with the information in the literature [37-40], we have found that PDGFRα up-regulation associated with MAPK and PI3K/AKT activation is regulated by the Shh pathway and by Gli1 activation. Our data confirm this relationship since treatment with BRAF-I enhances Gli1 expression. The latter results are associated with PDGFRα up-regulation mediated BRAF-I resistance since treatment with the novel clinically approved Shh-I LDE225 down-regulates the expression of PDGFRα by inhibiting Gli1 activation. Furthermore, PDGFRα down-regulation by the Shh-I LDE225 in combination with vemurafenib enhances tumor growth inhibition *in vitro* and *in vivo* and decreases ERK and AKT activation in both sensitive and resistant cell lines.

PDGFRα is not the only growth factor receptor which plays a role in BRAF-I resistance. IGFR1[14] and PDGFRβ[11, 19] are involved in the acquired BRAF-I resistance of melanoma. BRAF-I resistance induced by IGFR and PDGFRβ, similar to PDGFRα, is mediated by ERK and AKT activation. However as reported by others[19] and as found by us (data not shown) the PDGFRα/PDGFRβ inhibitors sunitinib and imatinib are not able to overcome BRAF-I resistance mediated by PDGFRβ up-regulation. The latter finding reflects the lack of inhibition of ERK activation in spite of the inhibition of AKT activation since the inhibition of these two downstream components of the RAF/MEK/ERK and PI3K/AKT signaling pathways by a PDGFRβ-specific shRNA restored sensitivity of melanoma cells to vemurafenib.

The potential clinical relevance of our results is suggested by two lines of evidence. First, PDGFRα expression was up-regulated in 5 out of the 9 matched melanoma lesions with a BRAF(V600E) mutation, surgically removed from patients who had developed BRAF-I resistance. Second, the extent of PDGFRα increase in melanoma lesions, as measured by the increase in the percentage of stained melanoma cells, was associated with the clinical course of the disease. Specifically a marked increase in PDGFRα expression was associated with a shorter time to progression and less tumor regression based on RECIST criteria. Notably, baseline expression of PDGFRα did not correlate with response or time to progression. In order to utilize the phenomenon we have observed as a method for patient selection, one would need to monitor PDGFRα up-regulation in tumor biopsy specimens or to develop a noninvasive or surrogate method to detect PDGFRα up-regulation.

The evidence we provide represents a strong rationale to translate to a clinical setting the combinatorial strategy we have shown to be effective in counteracting the BRAF-I Gli1/PDGFRα-mediated resistance of melanoma cells both *in vitro* and *in vivo*. The translation of this approach into the clinic is facilitated by the availability of Food and Drug Administration (FDA) approved drugs to use in combination. Furthermore, these data suggest that PDGFRα may be a useful biomarker to identify patients with BRAF-mutant melanoma who will or will not respond to BRAF-I or combination BRAF-I and MEK-I. Lastly PDGFRα up-regulation has therapeutic implications since BRAF(V600E) melanoma patients with PDGFRα up-regulation may potentially benefit from treatment with BRAF-I in combination with PDGFRα-I or Shh-I.

## METHODS

### Cell cultures

The parental BRAF(V600E) melanoma cell lines Colo38 and M21 were cultured in RPMI 1640 medium (Mediatech) supplemented with 2 mmol/L L-glutamine (Mediatech) and 10% fetal calf serum (FCS; Atlanta Biologicals). The cell lines M21 and Colo38 were originated by the late Dr. Donald Morton (when he was at the University of California) and by the late Dr. George Moore (when he was at University of Colorado), respectively, from metastatic lesions of patients with melanoma. The BRAF(V600E) melanoma cell line TPF-10-741 was cultured in DMEM (Mediatech) supplemented with 2 mmol/L L-glutamine and 10% FCS. This cell line was started from a cutaneous metastasis of the melanoma patient TPF-10-741 who had developed BRAF-I resistance following treatment with vemurafenib. Melanoma cell lines with acquired vemurafenib resistance (Colo38R and M21R) were generated by propagating parental Colo38 and M21 cells in increasing concentrations of BRAF-I (up to 2 μM). At the end of 2 months, resistant cells were isolated from each of the two cell lines and cultured in RPMI 1640 medium supplemented with 2 mmol/L L-glutamine, 10% FCS and 500 nM vemurafenib. All cells were cultured at 37°C in a 5% CO_2_ atmosphere.

### Chemical reagents, antibodies and shRNAs

Vemurafenib was purchased from ChemieTek. Sunitinib, imatinib, crenolanib and LDE225 were purchased from Selleck Chemicals LLC. MTT was purchased from Sigma. p-AKT (Ser473)-, AKT-, p-PI3K p85 (γ458)-, p-CRAF(S289/296/301)-, p-MEK 1/2 (S217/221)-, p-ERK 1/2 (Thr202/Tyr204)-, ERK1/2-, PDFGRβ-, p-PDGFRα-, PDGFRα-, PTEN-, VEGFR2-, Cleaved Caspase-3 (Asp175)-, p-Histone H3 (Ser10)-, Gli1- and β-actin-specific monoclonal antibodies (mAbs) were purchased from Cell Signaling Technology. The calnexin-specific mAb TO-5 was developed and characterized as described [55]. PDGFRα-specific shRNA and GFP-shRNA were provided by the- Vector Core Facility of the University of Pittsburgh Cancer Institute.

### Patient Samples

Patients with metastatic melanoma harboring the BRAF(V600E) mutation (confirmed by genotyping) were enrolled in clinical trials with the BRAF-I (vemurafenib) or with the BRAF-I (dabrafenib) and MEK-I (trametinib) combination. Patients were consented for tissue acquisition per institutional review board (IRB)-approved protocol. Tumor biopsies were performed pre-treatment (day 0), at 10-14 days on treatment, and/or at the time of disease progression as defined by Response Evaluation Criteria In Solid Tumors (RECIST) if applicable. Formalin-fixed tissue was analyzed to confirm that viable tumor was present via hematoxylin and eosin (H&E) staining.

### Cell proliferation and MTT assay

Cells were plated in triplicate in 96-well microtiter plates at the density of 2.5 x 10^3^ per well in 100ul of RPMI 1640 or DMEM medium supplemented with 2% FCS and treated with vemurafenib and/or PDGFRα-I (sunitinib, imatinib and crenolanib) and/or Shh-I LDE225. Dimethyl sulfoxide (DMSO) (vehicle of the drugs) concentration was maintained at 0.02% in all wells. Doses of drugs to be used in the combinatorial treatment were chosen based on their IC50 determined with the melanoma cell lines tested. Cell proliferation was evaluated at the indicated time points utilizing the MTT assay which was carried out as reported elsewhere [56]. Data are expressed as percent of inhibition or percent of proliferation of treated cells as compared to untreated control cells. All experiments were performed three independent times in triplicates.

### Western Blot analysis

For sample preparation from cell lines, cells were seeded at the density of 1 x 10^5^ per well in a 6-well plate in medium supplemented with 2% FCS and the indicated doses of each drug or their combinations at 37°C in a 5% CO_2_ atmosphere for up to 72 h. The DMSO (vehicle of the drugs) concentration was maintained at 0.02% in all wells. Untreated cells were used as a control. Cells were collected and lysed in lysis buffer [10 mM Tris–HCl (pH 8.2), 1% NP40, 1 mM EDTA, 0.1% bovine serum albumin (BSA), 150 mM NaCl) containing 1/50 (vol/vol) of protease inhibitor cocktail (Calbiochem). For sample preparation from tumor xenografts, tumors were harvested from the mice when they were sacrificed and stored at -80°C. Proteins were extracted by homogenization in the presence of 2 to 5 ml lysis buffer. Western blot assay for signaling-related proteins was carried out as described [57]. The investigator who analyzed the sample from tumor xenografts was blinded to the type of treatment received by the mice used as the source of the tumor.

### RT-PCR

Total RNA was isolated from melanoma cells using the RNeasy kit (Qiagen). Reverse transcription was performed using SuperScript II Reverse Transcriptase (Invitrogen) followed by qPCR using Fast SYBR Green Master Mix (Applied Biosystems). The following primers were used: NRAS fragment from 41-312 fwd:GCCGCATGACTCGTGGTTC rev:TCAGTGCGCTTTTCCCAACA; BRAF fragment from 1526-1934 fwd:GCACCTACACCTCAGCAGTT rev:TGACTTCTGGTGCCATCCAC. Sequences were aligned to the human reference sequence using the ClustalW2 v2.1 algorithm.

### Transduction of melanoma cells with Lentiviral vectors encoding shRNA

M21R and TPF-10-741 cells were seeded at the density of 6 x10^4^ per well in a 6-well plate and incubated in culture medium for 24 h at 37°C in a 5% CO_2_ atmosphere prior to viral infection. Cells were transduced with PDGFRα-specific shRNA lentiviral particles [Target sequence: CCAGCCTCATATAAGAAGAAA (#1), CCAGCTTTCATTACCCTCTAT (#2), CGGTGAAAGACAGTGGAGAT (#3), CCCAACTTTCTTATCCAACTT (#4), CAATGGACTTACCCTGGAGAA (#5)] (1 x 10^6^ per well) in presence of polybrene (2 μg/ml) as described elsewhere [58]. Cells transduced with GFP-shRNA were used as a control. Following an 18 h incubation at 37°C, culture medium was removed and replaced with fresh culture medium. Following an additional up to 72 h incubation at 37°C, cells were analyzed for GFP expression under the microscope, split, enriched for infected cells by selection with puromycin (2.5 ug/ml) and collected for further analysis.

### Immunohistochemistry

Patient biopsies and tumors generated in mice were formalin fixed and paraffin embedded and then used as substrates in immunohistochemical reactions. Five-μm thick xenograft tissue sections were fixed on silane-coated glass slides, deparaffinized, and subjected to antigen retrieval (Target retrieval solution, DAKO). Following blocking, slides from mice were incubated with Cleaved Caspase-3 (Asp175) and p-Histone H3 (Ser10) –specific mAbs overnight. Four-μm thick sections from patient-derived samples were fixed on silane-coated glass slides, deparaffinized, and subjected to antigen retrieval (Target retrieval solution, DAKO). Sections were then incubated with PDGFRα-specific mAb (sc-338, Santa Cruz) (1:400) overnight. All sections were then washed with PBS, and the primary antibody was amplified using the VECTASTAIN ABC Kit (Peroxidase rabbit IgG, Vector Laboratories, PK-4001). The detection of this antibody was performed with the DAB Peroxidase Substrate Kit from DAKO and the sections were counterstained with H&E. Tissue from a human GIST and its lymphocyte infiltrate were used as a positive and a negative control, respectively, for PDGFRα expression. PDGFRα expression, as measured by the percentage of stained melanoma cells, in tumors harvested from BRAF-I treated patients either on treatment or at the time of disease progression, was compared to that in pretreatment tumors. Scores were recorded semiquantitatively as 1+, 2+, 3+ and 4+, when , 1–25%, 26–50%, 51-75% and >75% of melanoma cells were stained, respectively. Mitotic and apoptotic tumor cells in the sections of primary tumors harvested from mice were detected by staining p-Histone H3 (Ser10) and Cleaved Caspase-3 proteins, respectively, and quantified by counting 5 random fields per section (magnification ×200). Data are expressed as the mean number of mitotic or apoptotic tumor cells in each group. The number of mitotic and apoptotic tumor cells was counted by an investigator who was blinded to the type of treatment received by the mice from which tumors had been harvested.

### Assessment by flow cytometry of apoptosis induction

Apoptosis was detected by cytometric staining performed as described [59]. Briefly, apoptotic cells were identified by staining with Annexin -V and propidium iodide (PI) (BD Bioscience) for 15 min at room temperature. Flow cytometry data were analyzed using Summit v4.3 software (DAKO).

### In vivo studies

C.B-17 SCID female mice (8–10 week old) were purchased from Taconic Farms, Inc. Parental and BRAF-I resistant cell lines M21 and M21R (1 x 10^6^ cells/mouse) were implanted subcutaneously in the right lateral flank of mice. A total of 20 SCID mice was challenged with each cell line. Body weight and tumor volume were measured twice per week. Tumor volume was measured by vernier caliper. Treatment was initiated 10 days after cell inoculation when the tumor developed and had a diameter of around 0.4 cm. Doses of sunintib and imatinib were chosen based on data available in the literature [26, 27, 31, 33]. To test vemurafenib and sunitinib combination, mice were randomly divided into 4 groups of 5 mice each. Mice in Group 1 were treated with vemurafenib (12.5 mg/kg/twice per day [60]), those in Group 2 with sunitinib (20 mg/kg/day)[22] and those in Group 3 with vemurafenib (12.5 mg/kg/twice per day) and sunitinib (20 mg/kg/day) combination. Mice in Group 4 were left untreated as a reference for the natural course of the disease. To test vemurafenib and imatinib combination, mice were randomly divided into 4 groups of 5 mice each. Mice in Group 1 were treated with vemurafenib (25 mg/kg/twice per day [60]), those in Group 2 with imatinib (100 mg/kg/day)[23] and those in Group 3 with vemurafenib (25 mg/kg/twice per day) and imatinib (100 mg/kg/day) combination. Mice in Group 4 were left untreated as a reference for the natural course of the disease. To test vemurafenib and LD225 combination, mice were randomly divided into 4 groups of 5 mice each. Mice in Group 1 were treated with vemurafenib (12.5 mg/kg/twice per day), those in Group 2 with LDE225 (40 mg/Kg/day) [41], and those in Group 3 with vemurafenib (12.5 mg/kg/twice per day) plus LDE225 (40 mg/kg/day). Mice in Group 4 were left untreated as a reference for the natural course of the disease. Drugs were administered by oral gavage. When a tumor from untreated mice reached the maximum diameter as approved by the Institutional Animal Care and Use Committee (IACUC) all mice were sacrificed. Primary tumors and organs were collected for further analysis. Animal studies have been approved by the IACUC.

### Statistical analysis

Averages, standard deviations, and unpaired *t*-test were calculated using MS-Excel. Data are shown as mean ± SD of the results obtained in at least three independent experiments. Time of disease progression (time to progression) of BRAF-I treated patients was studied using the Kaplan-Meier method and difference between groups was calculated using the log-rank test. Differences between groups were considered significant when the *P* value was < 0.05. The asterisk (*) indicates *P*<0.05.

## SUPPLEMENTARY FIGURES


