# Supplementary material for: PDGFRα up-regulation mediated by sonic hedgehog pathway activation leads to BRAF inhibitor resistance in melanoma cells with BRAF mutation
Source: Oncotarget. 2014 Mar 31;5(7):1926–41. doi: 10.18632/oncotarget.1878 (PMC4039118; doi:10.18632/oncotarget.1878)
Supplement: Supplementary file 1 [file oncotarget-05-1926-s001.pdf]

PDGFRα up-regulation mediated by Sonic Hedgehog pathway activation leads to BRAF inhibitor resistance in melanoma cells with BRAF mutation

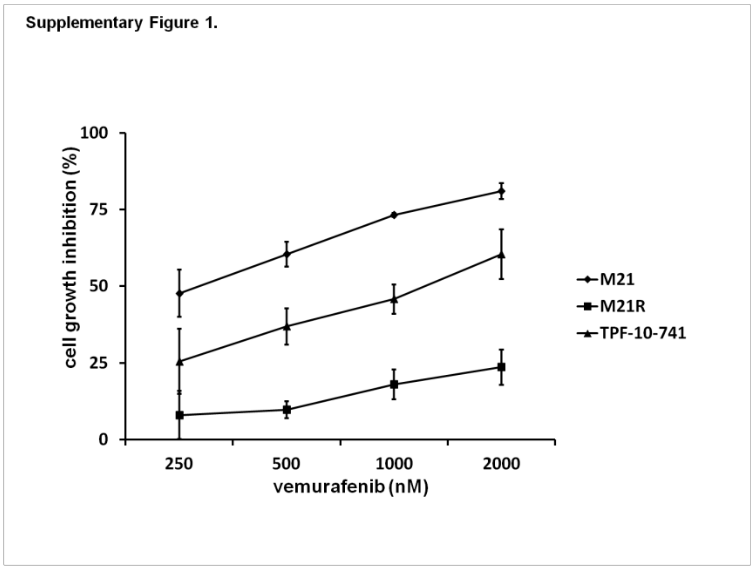

Supplementary Figure 1: Dose dependent effect of BRAF-I vemurafenib on the *in vitro* proliferation of BRAF-I sensitive and resistant melanoma cell lines harboring BRAF(V600E). M21, M21R and TPF-10-741 cells were treated with the indicated concentrations of the BRAF-I vemurafenib. Cell growth inhibition was determined by MTT assay following a 5 day incubation at 37°C. Percentage of cell growth inhibition was calculated as the ratio of treated to untreated cells for each vemurafenib dose. Data are expressed as the mean ± SD of the results obtained in three independent experiments.

Supplementary Figure 2.

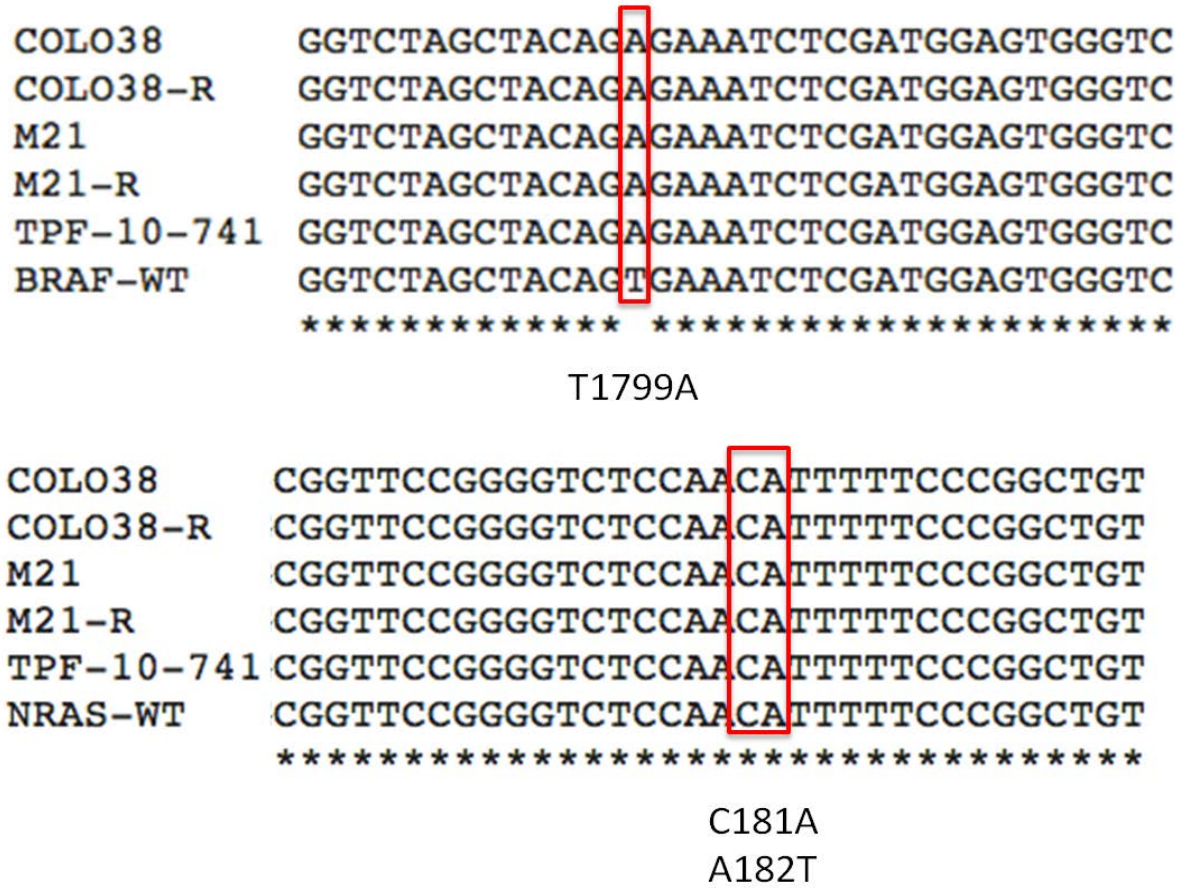

Supplementary Figure 2: Analysis of the BRAF and NRAS gene status in BRAF-I sensitive and resistant melanoma cells harboring BRAF(V600E). Total RNA was isolated from Colo38, Colo38R, M21, M21R and TPF-10-741. BRAF and NRAS gene sequences were analyzed utilizing RT-PCR. BRAF wild type (BRAF-WT) and NRAS wild type (NRAS-WT) genes were used as controls.

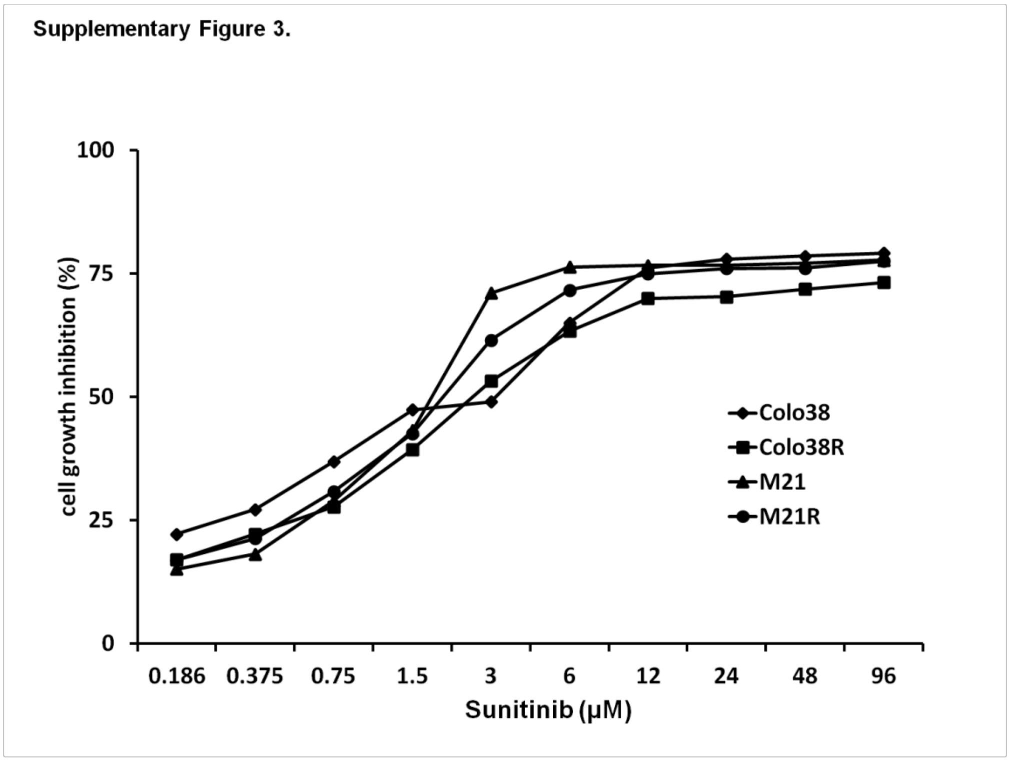

Supplementary Figure 3: Dose dependent effect of PDGFRα-I sunitinib on the *in vitro* proliferation of BRAF-I sensitive and resistant melanoma cell lines harboring BRAF(V600E). Colo38, Colo38R, M21 and M21R cells were treated with the indicated concentrations of sunitinib. Cell growth inhibition was determined by MTT assay following a 5 day incubation at 37°C. Percentage of cell growth inhibition was calculated as the ratio of treated to untreated cells for each sunitinib dose. Data are expressed as mean ± SD of the results obtained in three independent experiments.

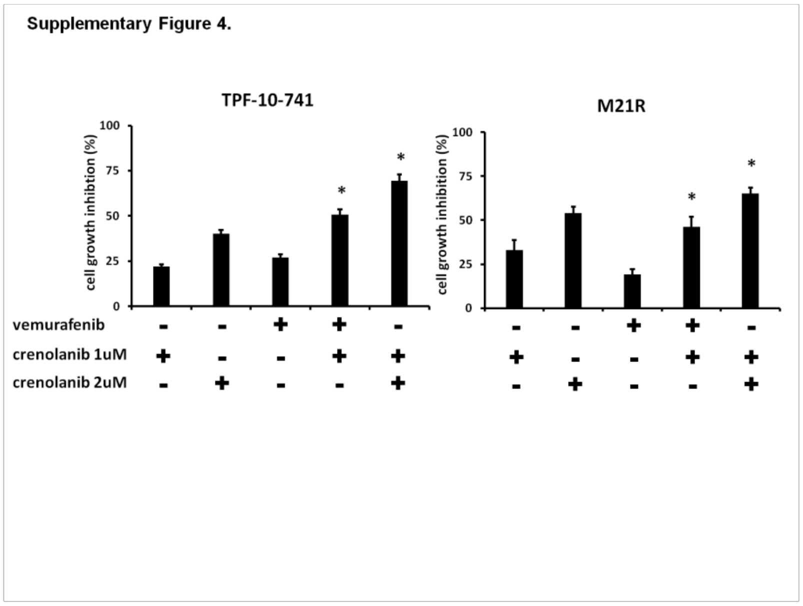

Supplementary Figure 4: Enhancement by PDGFRα-I crenolanib of the *in vitro* anti-proliferative activity of BRAF-I in BRAF-I sensitive and resistant melanoma cells harboring BRAF(V600E). M21R and TPF-10-741 cells were treated with the BRAF-I vemurafenib (500 nM) and/or the indicated concentrations of the PDGFRα-I crenolanib. Cell growth inhibition was determined by MTT assay following a 3 day incubation at 37°C. Percentage of cell growth inhibition was calculated as ratio of treated to untreated cells for each treatment. Data are expressed as mean ± SD of the results obtained in three independent experiments. The asterisk (*) indicates P<0.05.

Supplementary Figure 5.

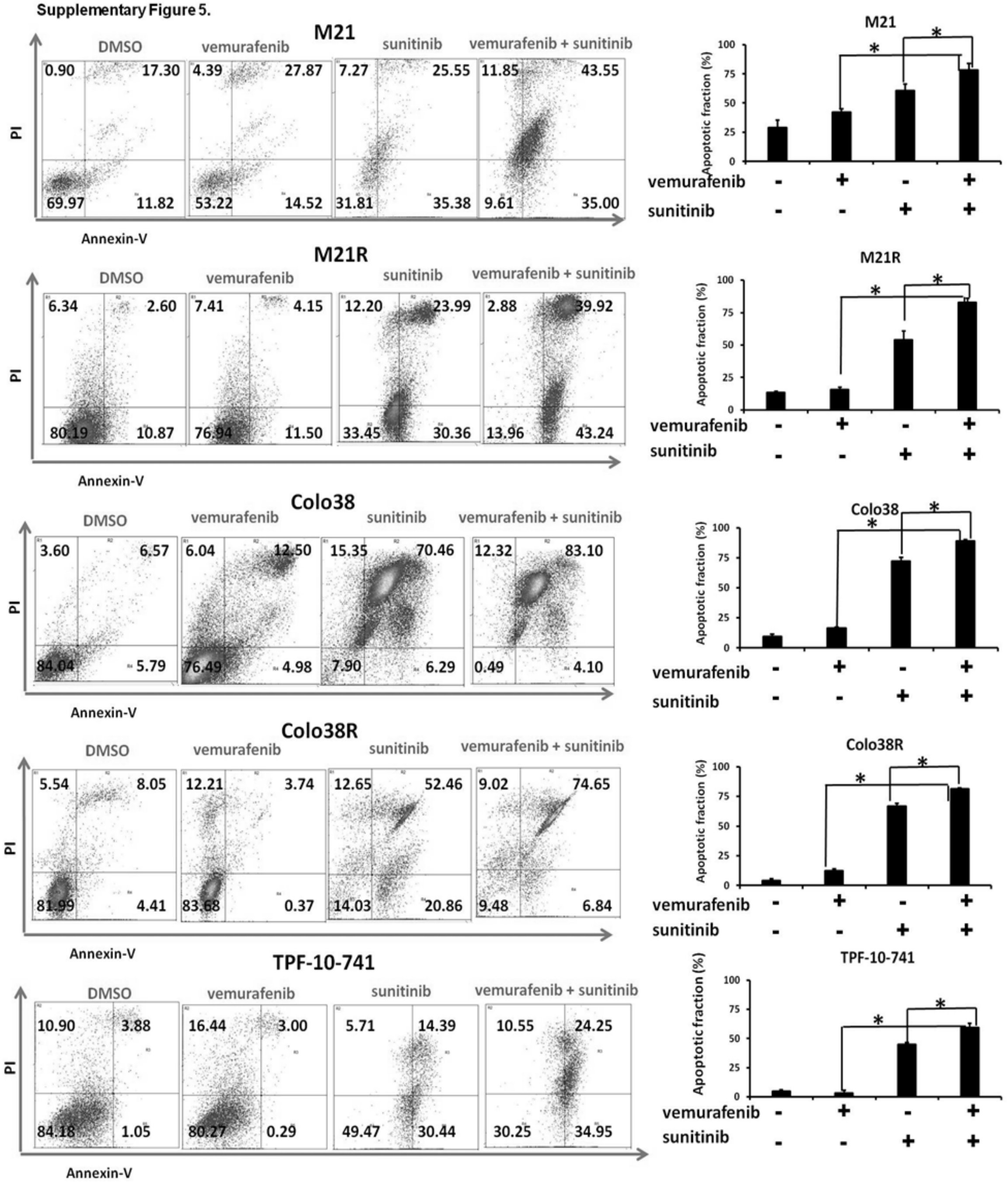

Supplementary Figure 5: Enhancement by PDGFRα-I of the *in vitro* pro-apoptotic activity of BRAF-I in BRAF-I sensitive and resistant melanoma cell lines harboring BRAF(V600E). M21, M21R, Colo38, Colo38R and TPF-10-741 cells were treated with the BRAF-I vemurafenib (500 nM) and/or the PDGFRα-I sunitinib (1.5 µM). Following a 24 h incubation at 37°C cells were harvested and stained with Annexin V and PI. A representative result is shown (left panel). The levels of apoptosis are plotted and expressed as mean fraction of apoptotic cells ± SD of the results obtained in three independent experiments (right panel). The asterisk (*) indicates $P<0.05$.

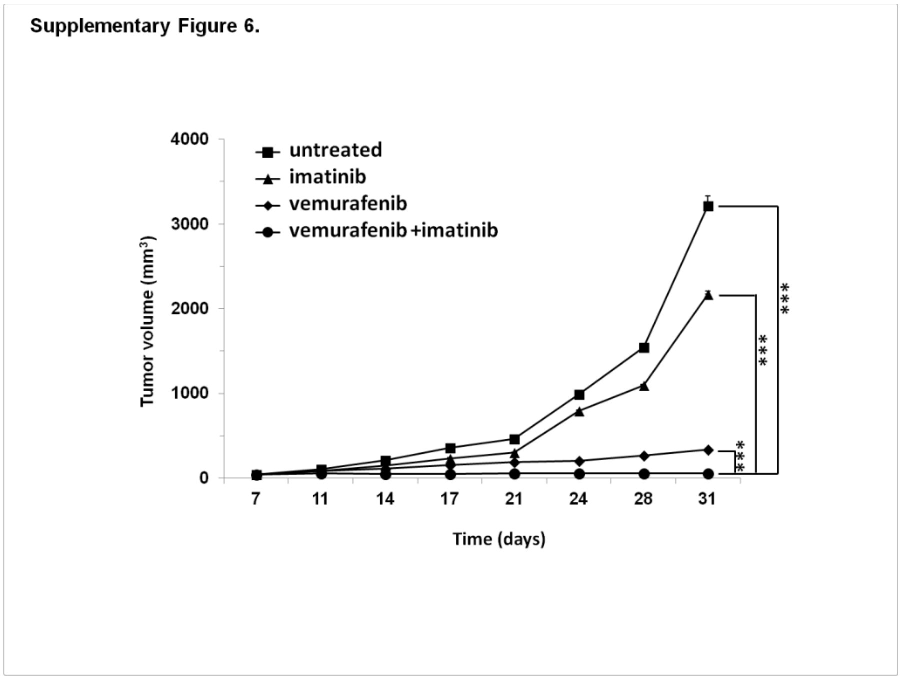

Supplementary Figure 6: Enhancement by PDGFRα-I imatinib of the growth inhibition by BRAF-I of BRAF(V600E) melanoma cells grafted in immunodeficient mice. M21 cells were implanted subcutaneously in 20 SCID mice. When tumors became palpable, mice were randomly divided into 4 groups (5 mice/group). One group was treated with the BRAF-I vemurafenib (25 mg/kg/twice per day), one with the PDGFRα-I imatinib (100 mg/kg/day) and one with vemurafenib (25 mg/kg/twice per day) in combination with imatinib (100 mg/kg/day). One group of mice was left untreated as a reference for the natural course of the disease. Efficacy data are plotted as mean tumor volume (in mm^3) ± SD. The asterisks (***) indicate $P<0.001$.
